# ARPES view on surface and bulk hybridization phenomena in the antiferromagnetic Kondo lattice CeRh_2_Si_2_

**DOI:** 10.1038/ncomms11029

**Published:** 2016-03-18

**Authors:** S. Patil, A. Generalov, M. Güttler, P. Kushwaha, A. Chikina, K. Kummer, T. C. Rödel, A. F. Santander-Syro, N. Caroca-Canales, C. Geibel, S. Danzenbächer, Yu. Kucherenko, C. Laubschat, J. W. Allen, D. V. Vyalikh

**Affiliations:** 1 Institute of Solid State Physics, Dresden University of Technology, Zellescher Weg 16, D-01062 Dresden, Germany; 2 Department of Physics, Indian Institute of Technology (Banaras Hindu University), Varanasi 221005, India; 3 Max IV Laboratory, Lund University, Box 118, 22100 Lund, Sweden; 4 CSNSM, Université Paris-Sud and CNRS/IN2P3, Batiments 104 et 108, 91405 Orsay, France; 5 Max Planck Institute for Chemical Physics of Solids, Nöthnitzer Str. 40, D-01187 Dresden, Germany; 6 European Synchrotron Radiation Facility, 71, Avenue des Martyrs, Grenoble 38000, France; 7 Institute for Metal Physics, National Academy of Sciences of Ukraine, Vernadsky blvd. 36, UA-03142 Kiev, Ukraine; 8 Randall Laboratory, University of Michigan, 450 Church St, Ann Arbor, Michigan 48109-1040, USA; 9 Saint Petersburg State University, Saint Petersburg 198504, Russia; 10 Donostia International Physics Center (DIPC), Departamento de Fisica de Materiales and CFM-MPC UPV/EHU, 20080 San Sebastian, Spain; 11 IKERBASQUE, Basque Foundation for Science, 48011 Bilbao, Spain

## Abstract

The hybridization between localized 4*f* electrons and itinerant electrons in rare-earth-based materials gives rise to their exotic properties like valence fluctuations, Kondo behaviour, heavy-fermions, or unconventional superconductivity. Here we present an angle-resolved photoemission spectroscopy (ARPES) study of the Kondo lattice antiferromagnet CeRh_2_Si_2_, where the surface and bulk Ce-4*f* spectral responses were clearly resolved. The pronounced 4*f* ^0^ peak seen for the Ce terminated surface gets strongly suppressed in the bulk Ce-4*f* spectra taken from a Si-terminated crystal due to much larger *f-d* hybridization. Most interestingly, the bulk Ce-4*f* spectra reveal a fine structure near the Fermi edge reflecting the crystal electric field splitting of the bulk magnetic 4*f* ^1^_5/2_ state. This structure presents a clear dispersion upon crossing valence states, providing direct evidence of *f-d* hybridization. Our findings give precise insight into *f-d* hybridization penomena and highlight their importance in the antiferromagnetic phases of Kondo lattices.

## Abstract (web summary)

In rare-earth intermetallics, interaction between localized 4*f* electrons and itinerant electrons can result in exotic states of matter. Here, the authors use photoemission spectroscopy to reveal and study this interaction in the bulk and at the surface of the Kondo lattice antiferromagnet CeRh_2_Si_2_.

## Graphical abstract

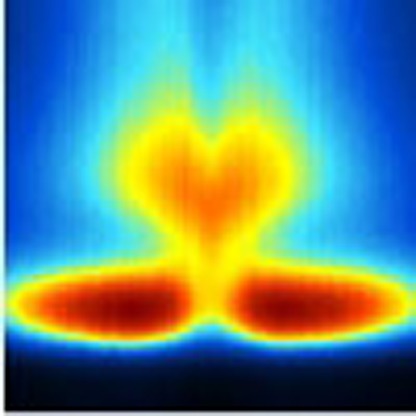

In intermetallics, Ce is close to the trivalent 4*f* ^1^(5*d*6*s*)^3^ configuration with slight to moderate 4*f* ^0^(5*d*6*s*)^4^ and 4*f* ^2^(5*d*6*s*)^2^ admixtures. The 4*f* ^2^ configuration with double *f* occupancy is rather unfavourable due to the strong onsite Coulomb repulsion compared to the 4*f* binding energy, and its contribution usually does not exceed a few percent. In contrast, the tetravalent 4*f* ^0^ lies energetically closer to 4*f* ^1^ which often leads to a fluctuating valence between both configurations. Therefore, the unusual phase diagrams in many Ce systems result from a competition between the non-magnetic 4*f* ^0^ and the magnetic 4*f* ^1^ ground state configuration with momentum *J*=5/2.

At the level of the Anderson impurity model with hybridization between 4*f* and valence band states, the competition is resolved through the Kondo quenching of the 4*f* ^1^ magnetic moment and the resulting appearance of the Kondo resonance at the Fermi energy (*E*_F_) in the single-particle spectral function. The resonance reflects final states with a predominantly 4*f* ^1^ contribution for both the electron removal and addition spectrum. In Kondo lattices, it can be understood as the momentum integrated coherent quasi-particle part of the single particle spectrum^1^. In a crystalline environment, the degeneracy of the Ce 4*f* ^1^_5/2_ sextet is lifted due to the interaction with the nonspherical electrostatic field of the ligands, which is called the crystal electric field (CEF). The resulting CEF splitting strongly influences the magnetic properties of the compound^2^. The Kondo lattice quasi-particles form heavy bands originating from the CEF split 4*f* ^1^ states. If the magnetic moments are Kondo quenched, then these bands disperse across *E*_F_ so that the 4*f* electrons enter the Fermi volume, resulting in the so-called large Fermi surface^1^,^3^,^4^,^5^,^6^. However, for a magnetically ordered material the heavy bands must be polarized. Depending on the model and the strength of the polarization, this is expected to modify the Fermi surface towards the small one^3^,^7^,^8^ in which the 4*f* electrons are excluded from the Fermi volume. Thus, in the case of large ordered moments, many low-temperature properties, like for example, the Fermi surfaces or the Sommerfeld coefficient, do not reflect any more the bare hybridization, because its effect is masked by the strong polarization. This results in some ambiguity in characterizing the nature of the 4*f* electrons in such systems^7^. The entanglement between 4*f* and valence states in such systems has not been deeply addressed in the past, but is now becoming an important and pressing issue, for example, because of the observation of metamagnetic transitions with huge changes in the Fermi surface in quite a number of compounds located in this regime, and with controversial interpretations (see for example, refs 9, 10).

CeRh_2_Si_2_ is a system where the ambiguity in the nature of the 4*f* electrons is particularly striking^11^. On one hand, its high antiferromagnetic ordering temperature^12^, *T*_N_≈38 K, being the third highest among Ce systems; its large ordered moment *m*_AF_≈1.4*μ*_B_ (refs 12, 13), and most especially its large entropy of nearly *R*ln2 at *T*_N_ (ref. 14) are strong evidence for a fully localized 4*f* electron. Accordingly, de Haas van Alphen (dHvA) experiments^15^ reported the observation of a small Fermi surface. On the other hand, other properties like for example, the width of the quasi-elastic line in inelastic neutron scattering^16^,^17^ imply a large Kondo scale of the order of *T*_K_∼30 K. Furthermore, the transition to a paramagnetic ground state at a comparatively small pressure *p*_c_≈1.1 GPa (ref. 18) indicates a close proximity of the 4*f* state to a magnetic instability. The large *T*_K_ and the small *p*_c_ indicate a significant hybridization of the 4*f* electrons. However, in CeRh_2_Si_2_ magnetism wins over Kondo at ambient pressure, and therefore the effect of this large hybridization on low-*T* properties gets masked, making a study and an assessment of this hybridization difficult.

Here we study the single particle spectrum of the Ce 4*f* electronic states in the antiferromagnetic (AFM) Kondo lattice CeRh_2_Si_2_ by means of high-resolution angle-resolved photoelectron spectroscopy (ARPES). Generally, the Ce 4*f* photoemission spectroscopy (PES) spectrum shows a characteristic double-peak structure with one peak at about 2 eV binding energy (BE) corresponding roughly to the 4*f* ^0^ final state configuration as expected from the ionization of the trivalent 4*f* ^1^ ground state, and a second feature at *E*_F,_ commonly ascribed to the tail of the Kondo resonance that is centered slightly above *E*_F_ (refs 1, 19). Theoretically, this spectral function is well-understood and well-described within the Kondo and Anderson lattice models. The ∼2-eV feature, which carries most of the 4*f* spectral weight, reflects the localized, ionization-like aspect of the Ce 4*f*, while the weaker feature at *E*_F_ reflects the hybridized, quasi-particle aspect. However, a precise experimental confirmation of all expected features faced strong problems connected with differentiating between surface and bulk electronic contributions^1^. So far, however, poorly defined surfaces have resulted in a mixture of surface- and bulk-related signals in ARPES, preventing a precise analysis and assignment. High-energy PES strongly enhances the bulk part of the signal, but the much poorer energy resolution prevents the detection of fine structures, in particular close to *E*_F_. Taking advantage of our expertize gained in the course of extended ARPES studies on the homologues YbRh_2_Si_2_ and EuRh_2_Si_2_, we were able to obtain spectra for the Ce-terminated and the Si-Rh-Si terminated surfaces of CeRh_2_Si_2_, which are representative for weakly and strongly hybridized 4*f* states, respectively. Comparing both spectra provides an unprecedented view into the effects of *f−d* hybridization on the electronic spectral function, revealing features such as a CEF induced fine structure near the Fermi edge for the Si−Rh−Si surface. For this surface, which is representative for bulk CeRh_2_Si_2_, we observe a strong interaction between valence and *f* electrons at a temperature well below *T*_N_, which demonstrates the importance of hybridization effects in the antiferromagnetic phases of Kondo lattices.

## Results

### Si- and Ce-surface termination of CeRh_2_Si_2_ viewed by ARPES

We begin with a general characterization of different surface terminations of cleaved CeRh_2_Si_2_ crystals. Our previous ARPES studies on structurally similar RET_2_Si_2_ materials (RE=Eu, Yb and T=Co, Rh, Ir) demonstrated that cleavage always takes place between Si and RE layers leaving behind either a RE or a Si terminated surface^20^,^21^,^22^,^23^,^24^. The covering of a RE plane with a Si−Rh−Si trilayer is usually sufficient to create a bulk-type chemical surrounding for the RE ions^25^,^26^. The Si-terminated surface of cleaved CeRh_2_Si_2_ may thus be used to evaluate the bulk contribution of the Ce 4*f* spectrum. For the Ce-terminated surface, however, the ARPES spectra are dominated by emissions from the outermost Ce layer and give information about the surface properties of Ce in CeRh_2_Si_2_. We also utilize two PES cross-section effects, a Fano resonance enhancement of the Ce 4*f* emission for photon energies around 121 eV, corresponding to the Ce 4*d*→4*f* X-ray absorption edge^1^, and a Cooper minimum suppression^21^ of the Rh 4*d* emission, coincidentally also around 121 eV.

**Figure 1:**
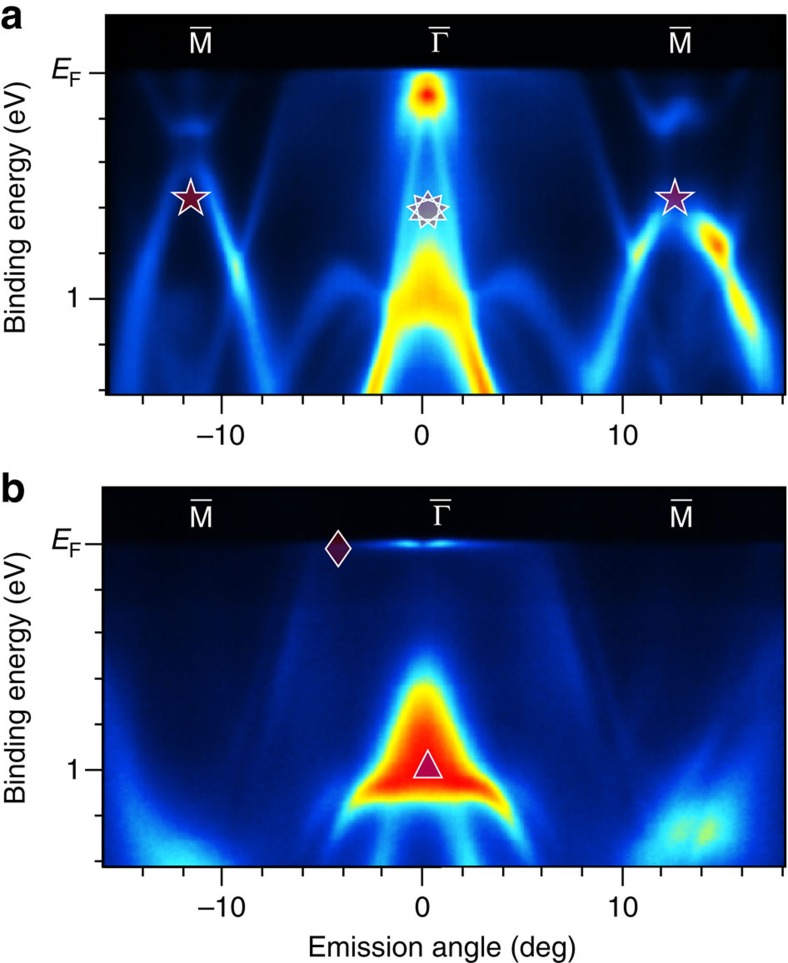
ARPES characterization of different surface terminations. ARPES data of (**a**) Si and (**b**) Ce terminated surfaces of CeRh_2_Si_2_ are shown in a 2D view where yellow (red) denotes the largest intensity. The data along the 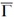
–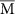 direction were taken at 112 eV photon energy (off resonance) and reveal mainly contributions from *spd*-derived bands. For the meaning of the symbols see the main text.

Figure 1a,b shows two off-resonance ARPES-derived band maps taken from a freshly cleaved CeRh_2_Si_2_ crystal close to the 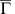
–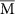 direction. These data reflect the discussed surface configurations: Si and Ce terminations can be distinguished by the presence/absence of a Shockley-type surface state labelled by star symbols. This state emerges within a huge gap in the projected bulk band structure centered at the 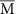
- point and is an intrinsic feature of the Si surface. Note that this spectral feature stems mainly from Si 3*s*, 3*p* and Rh 4*d* hybrid states, and was the focus of our earlier studies of the unusual ferromagnetic properties of the Si surface in EuRh_2_Si_2_ (ref. 24). Located within the topmost Si−Rh−Si trilayer, this surface state is missing in the Ce-terminated case, where the respective electrons participate in chemical bonds with the topmost Ce layer. In contrast to EuRh_2_Si_2_, the 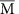
- point Shockley state does not seem to split down to a temperature of ∼1 K, even though the system is in its AFM phase^27^,^28^,^29^. This might be a consequence of the robust in-plane AFM order of the Ce moments along each Ce layer—in contrast to the ferromagnetic arrangement of the 4*f* moments within the Eu planes in EuRh_2_Si_2_—canceling the exchange interaction between the Ce moments and the electrons in the surface state. A further signature of differing surface terminations shows up in a hole-like, linearly dispersing surface resonant band labelled by a sun-symbol around the 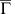 point in the Si-terminated case^22^,^23^, which is replaced by an intense, rocket-shaped feature (labelled by a triangle) below ∼0.5 eV BE for Ce termination. The Dirac cone (sun-symbol) arises from Rh 4*d* states in the topmost Si−Rh−Si trilayer and has similarly been found in YbRh_2_Si_2_ and EuRh_2_Si_2_ (refs 21, 22, 23). Note also that for the Ce-terminated surface we detect the fine structure (marked by a diamond symbol) close to *E*_F_, which will be discussed below.

### 4*f* spectrum for Ce atoms at the surface and in the bulk

**Figure 2:**
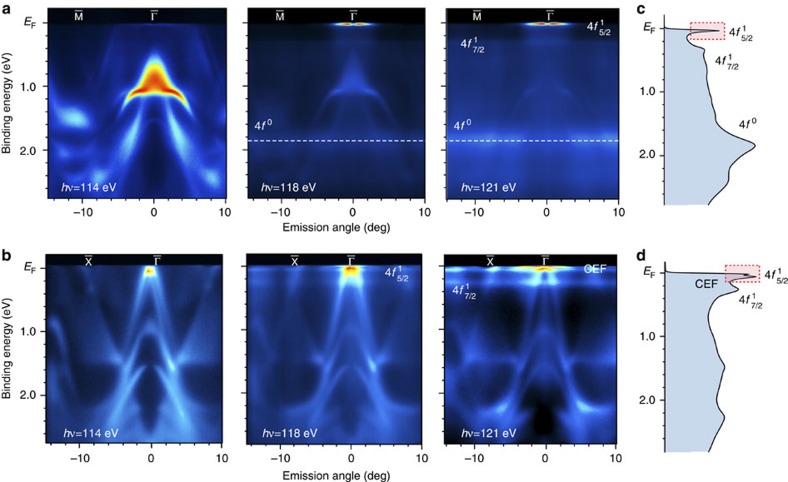
Insight into the Ce 4*f* spectral evolution for Ce- and Si-terminated CeRh_2_Si_2_. ARPES-derived band maps for (**a**) a Ce-terminated surface along 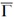
–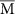 and (**b**) a Si-terminated surface along 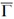
–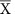 directions are shown taken at photon energies of 114, 118 and 121 eV. The data illustrate the evolution of momentum resolved Ce 4*f* emissions across the Ce 4*d*–4*f* threshold. Angle-integrated spectra taken at 121 eV photon energy for both surfaces are presented in **c**,**d**. Dotted rectangles draw attention to the 4*f*^1^_5/2_ peaks.

Tuning the photon energy towards 121 eV, strong contributions from Ce 4*f* emissions are expected. A series of three spectra ranging from off to on resonance for each surface termination, respectively, is shown in Fig. 2. For Ce termination (Fig. 2a), emission from Rh 4*d* states dominating the off resonance spectra is strongly suppressed by the Cooper minimum and replaced by the resonantly enhanced Ce 4*f* emission at 121 eV photon energy. The non-dispersive structure at about 1.9 eV BE and the flat spectral feature close to *E*_F_ are intensified indicating their Ce 4*f* origin. For Si termination (Fig. 2b), Rh 4*d* emission is similarly reduced, but now three flat bands appear right below *E*_F_, which did not show in the off resonance spectrum. These flat bands can be attributed to 4*f* emission from the bulk-type Ce layer buried beneath the Si−Rh−Si covering. The fine structure of the bulk Ce 4*f* emission consists of a spin-orbit side band at∼0.3 eV BE corresponding to the Ce 4*f* ^1^_7/2_ final state^1^ and weakly dispersive bands packed within tens of meV below *E*_F_.

The aforementioned spin-orbit sideband shows up for Ce termination as well. The intense 4*f* feature at *E*_F_, however, does not show a similar splitting as observed for the Si termination. The latter is nicely seen in the angle-integrated spectra shown in Fig. 2c,d, obtained by summing up the **k**-distributed spectral weight of the on-resonance data. These features lie within the dotted rectangles. As will be discussed below, the splitting seen in the Si-terminated data is the CEF splitting of the Ce 4*f* ^1^_5/2_ sextet under *D*_4h_ symmetry. It is interesting to note that large intensities of the *E*_F_ peak are restricted to a narrow region around the 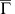
point at the Ce-terminated surface. A similar, but less pronounced, phenomenon is also observed for Si-termination. Large intensity of the *E*_F_ peak denotes strong *f*–*d* hybridization and the latter is particularly large at points in **k** space where valence bands approach or even cross *E*_F_ (refs 21, 22). This is nicely reflected in the data for the Si-terminated surface where the *E*_F_ peak intensity increases just below the *E*_F_ crossing of a band close to the 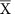 point. The large intensities around the 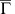 point may, thus, be attributed predominantly to hybridization with the apex of the Dirac cone. We note that the apparent sizeable *f* ^1^ spectral weight in the ‘bulk' spectra does not correspond to a large departure of the expectation value of the 4*f* occupancy *n*_f_ from the integer value 1. X-ray absorption spectroscopy measurements, which provide the most reliable information, determine an *n*_f_ of the order of 0.975, but this small decrease in *n*_f_ is typical for systems close to the transition from localized to itinerant 4*f* states, as for example, in CeCu_2_Si_2_ with *n*_f_=0.97 (ref. 17).

The broader peak emerging at ∼1.9 eV BE for Ce termination deserves particular attention. It arises from pure charge excitations of the trivalent Ce ion (4*f* ^1^→4*f* ^0^), and is usually referred to as the ionization peak^30^,^31^. A shoulder of this peak observed at 2.4 eV BE in the **k**-integrated spectrum reflects hybridization spreading due to structure in the valence band density of states. For Si termination, the intensity of the ionization peak is strongly reduced relative to the emissions close to *E*_F_ and consists of a broad bump at ∼1.5 eV BE, again with a shoulder due to hybridization spreading. The large intensity of the Fermi level peaks with respect to the ionization peak is indicative of strong *f*–*d* hybridization, much larger than that in the Ce surface layer, and is also reflected by the dispersive properties of the ionization peak in the **k**-resolved data. The larger BE of the leading ionization peak for the Ce-terminated surface is due to the surface-core-level shift, which is on the order of 0.4 eV for trivalent rare-earth systems, in good agreement with the shift of the peak from 1.5 to 1.9 eV observed here. Interestingly, the difference in the ionization peak signal between the weakly hybridized surface spectra and the stronger hybridized bulk spectra is very similar to the difference predicted for the *f* states within LDA+DMFT between CeFeAsO (weak hybridization) and CeFePO (strong hybridization)^32^.

**Figure 3:**
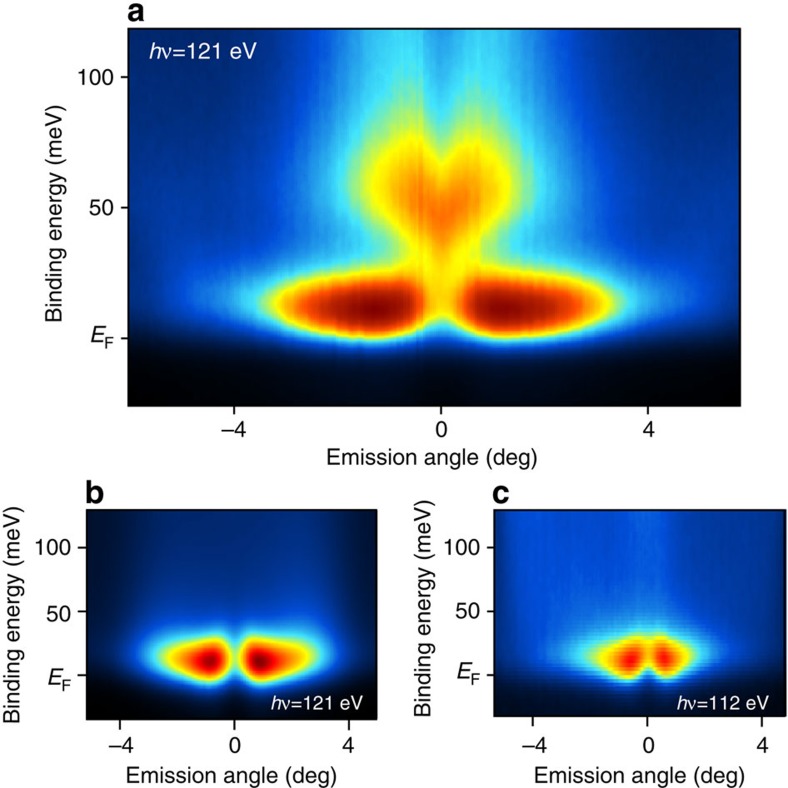
Fine electronic structure for Si and Ce surface terminations. ARPES spectra of the Ce 4*f* -derived states close to the E_F_ and near the 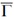 point taken in on-resonance for (**a**) Si and (**b**) Ce terminated surfaces are shown. ARPES data taken at Ce terminated surfaces in *off* -resonance are shown for comparison (**c**). The heart-shaped feature results from the hybridization of the CEF split Ce 4*f* states and the apex of the Dirac cone.

In Fig. 3, we take a closer look at the fine Ce 4*f* spectral structure near the 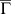-point. For the Si-terminated surface (Fig. 3a), two prominent features can be seen. An intense and weakly dispersive band bending down to ∼20 meV BE near the 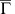
- point and a heart-shaped feature, which indicates hybridization of the Dirac cone apex with CEF 4*f*s running parallel to *E*_F_ and packed in a ∼50 meV range (Fig. 2b). The weakly dispersive band close to *E*_F_ is similarly found for Ce termination as shown in Fig. 3b except that the heart feature is missing. Since CeRh_2_Si_2_ is AFM ordered at the experimental temperature, we interpret non-zero BE as a characteristic feature arising due to the magnetically ordered ground state of the Ce 4*f* electrons^33^. Although appendix F in ref. 33 treats a ferromagnetic ground state, the underlying concept for a Kondo peak away from *E*_F_ should be valid also for an AFM. Note that the magnetically ordered ground state is separated from the Kondo resonant state by an energy corresponding to the stabilization of the magnetic state (*T*_N_∼3 meV for CeRh_2_Si_2_). Consequently, a PES feature appears just below *E*_F_ carrying a large weight due to the degeneracy of the Ce 4*f* orbital^33^.

In the off-resonance spectrum (Fig. 3c), where 4*f* emission is suppressed and the spectrum is dominated by valence-band emission, a similar feature is visible. This indicates the important role of Rh valence-band states in the formation of the fine spectral structure close to *E*_F_. Similar dispersions and *E*_F_ crossings of 4*f*-derived quasiparticle bands have already been observed in YbRh_2_Si_2_ (ref. 22) and CeFePO as well^34^.

### ARPES insight into the CEF splittings of 4*f* ^1^ state

Let us turn now to the discussion of the 4*f* CEF splittings detected by ARPES. We first note that according to standard theoretical modelling of PES on Kondo lattices^1^, the physics behind the CEF split lines in Ce- systems is quite different from that in Yb systems: In Yb-compounds, the 4*f* ^13^ final state is predominantly a result of a direct ionisation of a 4*f* ^14^ configuration admixed to the 4*f* ^13^ ground state. The amount of this admixture could be estimated from the relative intensity of the 4*f* ^13^ emission normalized to the whole 4*f* emission, and a more precise calculation within the Anderson model deviates only slightly from this result. In the Ce-compound, however, the admixture of 4*f* ^2^ configurations to the ground state are rather small, and the 4*f* ^1^ emission originates mostly from strong hybridization of 4*f* ^1^ and 4*f* ^0^ configurations. Thus, while in the Yb-system the intensity of the crystal-field PES signal is governed by the atomic dipole matrix elements, in Ce-systems its observability reflects its participation in the hybridization process. Accordingly, if the CEF states were not hybridized, then the photoemission spectral function would still show CEF satellites for an Yb valence fluctuating system, but no satellites for a homologue Ce system. Therefore, the observability of the CEF split state in CeRh_2_Si_2_ provides on its own a direct evidence for the hybridization of the excited CEF levels.

**Figure 4:**
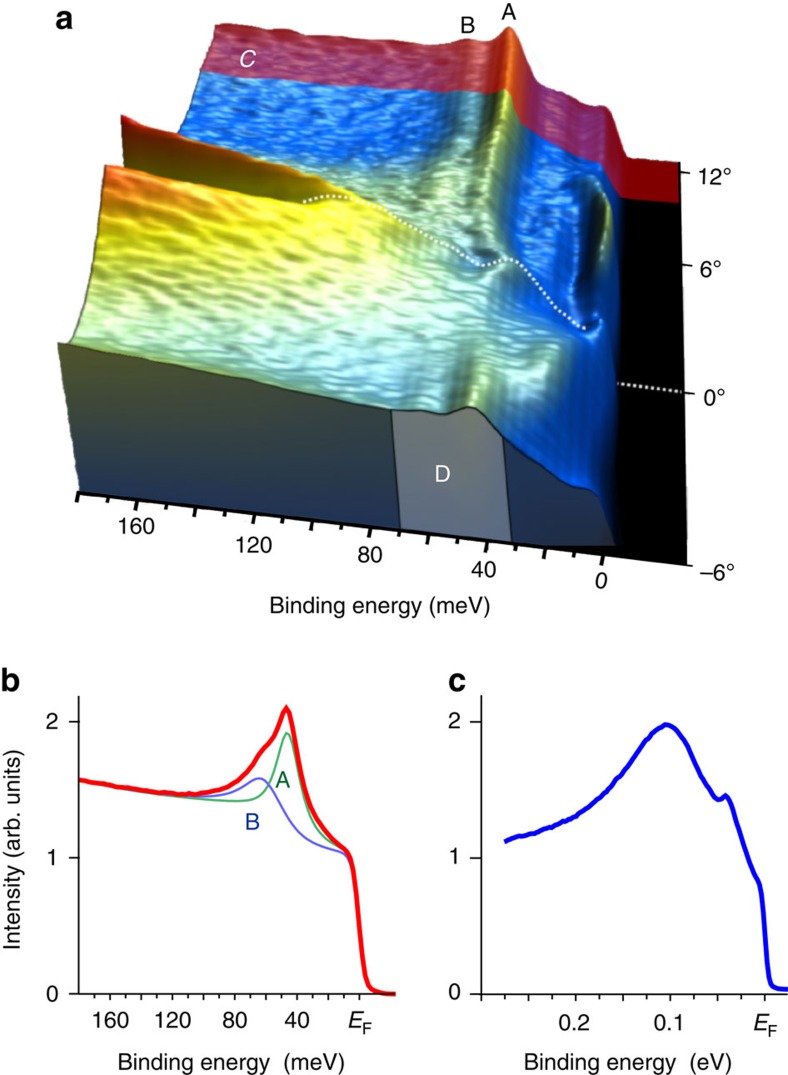
CEF splitting of the Ce 4f state and the hybridization phenomenon. (**a**) The ARPES data taken from the Si-terminated surface of CeRh_2_Si_2_ using 40 eV photons. Note that asymmetry of the cross-sections with respect to the 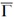 point is caused by the use of circularly polarized light. Each ARPES spectrum was normalized to the region labelled D between 30 and 70 meV BE, so that the integrated intensity for each energy distribution curve (EDC) is equal in this range. The line spectrum (**b**) was obtained by angle integration of EDCs within the shaded region labelled C. Its fit allows to deduce the CEF scheme: 0–48–62 meV. Note the fine dispersions of the CEF states visible between E_F_ and 50 meV. The ARPES spectrum taken at the 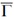 point marked as a dotted line in (**a**) is shown in (**c**).

In Fig. 4a, we show the ARPES data taken at 40 eV photon energy using circularly polarized light from the Si-terminated surface of CeRh_2_Si_2_. These experimental conditions allow us to clearly detect both 4*f* and valence band emission together, while still giving good contrast between them. Thus, we can conclusively identify the **k** space regions where the CEF states reveal their dispersive and non-dispersive behaviours. For each **k** value the spectra were normalized to constant integrated emission intensity in the BE range from 30 to 70 meV. We also show the **k**-integrated spectrum taken from region C. The ARPES data clearly show strongly dispersing hole-like bands, which stem mainly from Rh 4*d*-derived states. Also, we can see two states A and B that run parallel to *E*_F_ and reveal weak dispersions at points in **k** space where the valence bands approach them and hybridize. Apparently, the mentioned peak A at 48 meV and peak B at 62 meV BE reflect the fine structure of the 4*f* states. Approaching the 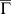
- point the component A disperses towards lower BE, due to its interaction with the Rh hole-like band, reaching about 30 meV exactly at 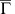, while component B seems to disperse in the opposite direction. The appearance of two components is in accordance with the expected CEF splitting in the tetragonal environment of *D*_4h_ point group symmetry, where the ground state Ce 4*f* ^1^_5/2_ splits into three Kramers doublets. Note that the ARPES data show certain *f*–*d* hybrid states that are packed between the ground state (0 meV) and the first excited CEF state (48 meV). Because the CEF split states have identical degeneracy, one may anticipate that the integrated spectral weight of all the CEF split states should be same. However, from Fig. 4 it can be seen that the feature A is more intense as compared with the other states. An apparent explanation for this difference is that the feature B and the *f*–*d* hybrid states below 48 meV are connected and that their strong dispersion due to hybridization with Rh 4*d* redistributes their spectral weights over a much larger energy window than that of feature A. Thus, the fine structure that we observe in the ‘4*f* ^1^' spectra indicates that one of the excited CEF doublets is strongly hybridized, while the other one is less hybridized. Notably, a recent theoretical calculation predicts the hybridization for the Γ_6_ doublet in CeRh_2_Si_2_ to be much weaker than for the Γ_7_ doublets^35^. Since all present experimental and theoretical studies indicate one of the Γ_7_ to be the CEF ground state and Γ_6_ to be at a comparatively high energy, all these observations can then be merged in a picture with a weakly hybridized Γ_6_ at 48 meV and a strongly hybridized Γ_7_ distributed in a wide energy range up to about 65 meV. Previous studies of the CEF scheme are far from being conclusive. Early determinations based on the temperature dependence of the magnetic susceptibility have suggested CEF level schemes of 0–32–80 meV (ref. 27) and 0–27–59 meV (ref. 28) with an almost pure |+/−5/2〉 CEF ground state. The most recent study is based on inelastic neutron scattering (INS) and X-ray-absorption spectroscopy^17^ and proposes a level scheme of 0–30–52 meV with a strong mixing of |+/−5/2〉 and |−/+3/2〉 in the ground state, which is incompatible with the measured susceptibility and magnetization data^27^,^28^. While INS is usually a proven technique to determine the CEF excitation energies, in CeRh_2_Si_2_ it revealed a very broad response, extending up to 60 meV, without well-resolved peaks, making the separation between phonon and magnetic excitations far from evident^17^. This broadness of the CEF response in INS is in line with the wide spectral distribution observed for the feature B and the *f*–*d* hybrid states in the PES 4*f* ^1^ signal. The PES results provide direct insight into the origin for this broadness, namely a strong hybridization with the Rh 4*d* states. On the other hand, assuming the A features in PES to correspond to the Γ_6_ doublet, we note that the INS cross section for the transition from a dominantly |+/−5/2〉 CEF ground state to the Γ_6_ excited state is comparatively small, thus the sharper peak expected in INS for this transition might be absorbed within the broad response related to the Γ_7_–Γ_7_ transition.

**Figure 5:**
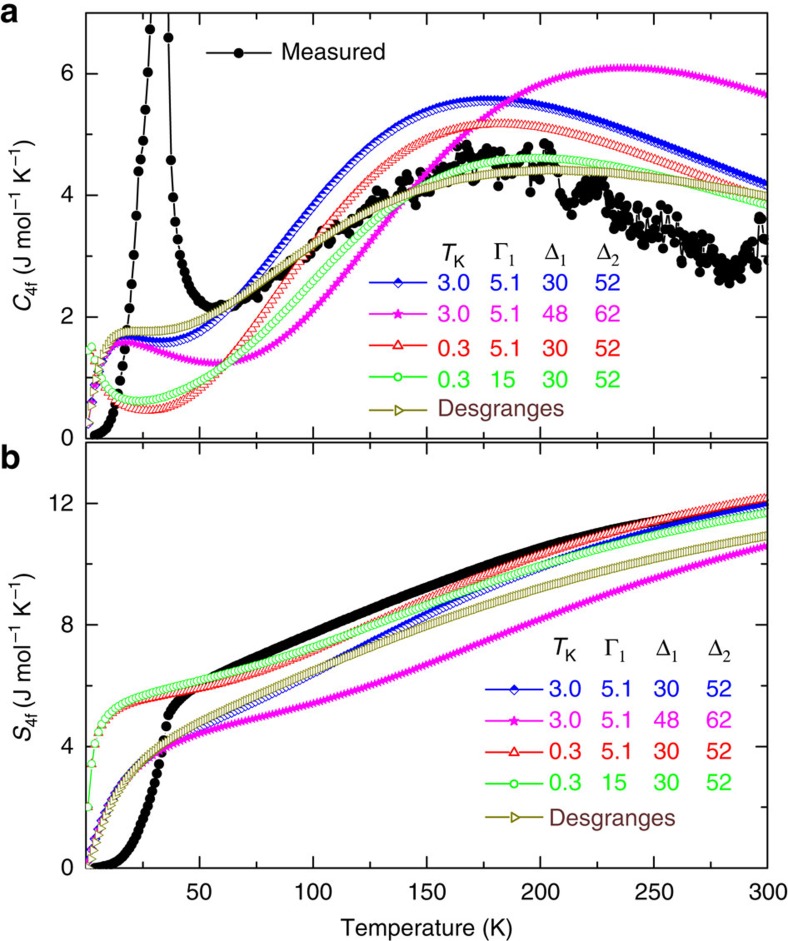
Insight into the specific heat and entropy of the Ce 4*f*. Temperature dependence of the 4*f* specific heat (**a**) and of the 4*f* entropy (**b**) in CeRh_2_Si_2_. Black dots show the values deduced from experiment. Coloured lines show the curves calculated for different values of the CEF splitting Δ_1_ and Δ_2_, of the Kondo scale T_K_, and of the width Γ_1_ of the first excited CEF level, using the model of Romero *et al*.^37^ or the numerical results of Desgranges^38^. This approach does not include AFM ordering, therefore, comparison with experiment is only meaningful for *T*>50 K. Parameter values are given in meV in the figure. Blue diamonds: values as deduced from INS. Magenta stars: same as before, but with Δ_1_ and Δ_2_ taken from our PES results. Red triangles: Parameter as deduced from INS, but with *T*_K_ reduced by a factor of 10 to account for the observed entropy at 50 K. Green circles: same as before, but with Γ_1_ increased by a factor of 3 in an attempt to reproduce the observed large Cp(T) for 40<*T*<100 K despite a low T_K_. Brown triangles: scaled numerical results of Desgranges^38^. It corresponds to the case Δ_1_=35 meV, Δ_2_=70 meV and *T*_K_=2.6 meV.

An established method to check the relevant energy scales of a Kondo lattice, that is, *T*_K_ and the CEF splitting, is to study the evolution of the 4*f*-specific heat *C*_4*f*_ (*T*) and 4*f* entropy *S*_4*f*_ (*T*) as a function of *T* (refs 36, 37). Usually one gets reasonable agreement between the experimental *C*_4*f*_ (*T*) and that calculated using parameters obtained from other methods. As an example, we could recently very nicely reproduce the experimental *C*_4*f*_ (*T*) of YbIr_2_Si_2_ using the CEF splitting deduced from the ARPES spectra, and thus demonstrate that INS missed the highest CEF level^26^. Therefore, we determined *C*_4*f*_ (*T*) and *S*_4*f*_ (*T*) of CeRh_2_Si_2_ in a wide *T* range, up to 300 K, and compared with the curves expected for different CEF schemes and different values of the Kondo temperature *T*_K_ (Fig. 5)^38^. There is presently no analytical expression available for the specific heat of a Kondo lattice which includes both AFM order and the Kondo effect on the excited CEF levels. Since in the present study we are specifically interested in the excited CEF levels, we focus on *C*_4*f*_ (*T*) and *S*_4*f*_ (*T*) above *T*_N_. In the standard way of modelling *C*_4*f*_ (*T*) the contribution of the excited CEF level is accounted for by a simple Schottky expression and thus broadening of the CEF levels due to the Kondo effect is not taken into account (see section methods). Since in CeRh_2_Si_2_ INS results evidence a significant broadening of the CEF levels, in line with the present PES results, we preferred to use the recent model of Romero *et al*.^37^, where the broadening of the first excited level can be taken empirically into account. We initially performed two calculations, one with the CEF excitation energies Δ_1_ and Δ_2_ as deduced from INS^17^, and one with Δ_1_ and Δ_2_ as deduced from the present ARPES results. In both cases we took *T*_K_ and width Γ_1_ of the first excited CEF level as deduced from INS^17^. Including AFM order would modify *C*_4*f*_ (*T*) and reduce *S*_4*f*_ (*T*) only in the *T* range from slightly above *T*_N_ to *T*=0. Above *T*_N_, the agreement between the experimental curve and the curve for the CEF scheme deduced from INS is not too bad, while the curve for the ARPES deduced CEF scheme is too much shifted towards high temperatures. However, in both cases the maximum connected with the CEF excitations is much narrower in the theoretical *C*_4*f*_ (*T*) curves than in the experimental ones, suggesting that the width of the CEF level has been underestimated in the analysis of the INS data. A more fundamental problem is that in both cases the calculated 4*f* entropy in the *T* range 50 K<*T*<100 K is significantly smaller than the experimental one. In an attempt to resolve this discrepancy, we first reduced *T*_K_ by one order of magnitude. The entropy above *T*_N_ is now nicely reproduced, but *C*_4*f*_ (*T*) is now severely underestimated in the *T* range 50 K<*T*<100 K. Increasing the width of the first excited CEF doublet does not correct this discrepancy. Furthermore such a low *T*_K_ is incompatible with many other properties, for example, the width of the quasi-elastic line in INS and strong hybridization seen in the bulk ARPES signal.

The specific heat of a Kondo ion including CEF splitting (with same hybridization strength for ground and excited CEF states) can be calculated exactly in the single ion case, but this requires very demanding numerical calculations which do not allow for ‘fitting' experimental data. Fortunately, Desgranges^38^ very recently provided such numerical results for a larger number of cases, and therefore we tried to use them for modelling our experimental *C*_4*f*_ (*T*) data. In this approach, one has first to choose the most appropriate case among those considered in ref. 38, and then to fix the only free parameter which is the Kondo scale in the absence of CEF splitting, *T*_KN6_. A preliminary survey of the results in ref. 38 suggests that the curve for Δ_1_=1/2̇Δ_2_=0.7·*T*_KN6_ is closest to our experimental data. Matching experimental and calculated *C*_4*f*_ (*T*) in the *T* range 50<250 K resulted in *T*_KN6_=50 meV. This corresponds to a Kondo scale *T*_K_=2.6 meV for the ground-state doublet and to CEF splitting of Δ_1_=35 meV and Δ_2_=70 meV. The agreement between calculated and experimental specific heat data in the *T* range 50 K<*T*<250 K is excellent (Fig. 5). A slightly poorer agreement is obtained for the case Δ_1_=Δ_2_=*T*_KN6_=43 meV (not shown), corresponding to *T*_K_=2.3 meV. However, in both cases the calculated entropy in the *T* range 50 K<*T*<100 K is significantly smaller than the experimental values, reflecting the same problem as for the fits with the model of Romero *et al*. The essence of this problem is that in the case of a CEF ground-state doublet well-separated from the excited CEF states due to a large splitting as in CeRh_2_Si_2_, the large Kondo interaction implied by other properties, like for example, the broadening of the CEF contribution to *C*_4*f*_ (*T*) or the (bulk) ARPES response, always reduces the entropy at 40 K significantly below *R*ln2. Resolving this problem requires considering further interactions beyond the single-ion Kondo and the CEF splittings.

Despite this intricate problem, the analysis of *C*_4*f*_ (*T*) supports our analysis of the bulk part of the ARPES signal. The broadness of the CEF contribution to *C*_4*f*_ (*T*) at high *T* implies a strong hybridization of the bulk 4*f* states, in line with our conclusion from the bulk PES signal, and in line with other properties. This shows CeRh_2_Si_2_ to be strongly hybridized even though it has fully localized 4*f* electrons in the sense of the large size of the AFM ordered moment, the large entropy at *T*_N_, and the small Fermi surface deduced from dHvA experiments^15^. The comparison of experimental *C*_4*f*_ (*T*) with exact numerical results for the Kondo+CEF single-ion model indicates the broadening of the excited CEF level to be even larger than the value deduced from INS results, but of the size expected when the hybridization of the CEF ground and excited states are identical. This suggests that in previous similar studies the broadening of the excited CEF states might have been underestimated. On the other hand, the analysis of *C*_4*f*_ (*T*) is not conclusive with respect to the exact CEF splitting, but supports a large value of this splitting, of the size we observed in the bulk ARPES signal.

In summary, we have presented an ARPES study of the antiferromagnetic Kondo lattice CeRh_2_Si_2_ which provides precise insight into how the hybridization between valence and *f* electrons affects the Ce 4*f* PES response. We discern two well-defined and different types of spectra, which we can clearly connect with the Si- and Ce-terminated surfaces of CeRh_2_Si_2_. The latter, which is representative for weakly hybridized Ce, shows a strong and sharp 4*f* ^0^ peak at ∼1.9 eV BE, and a structureless peak at the Fermi energy. In the Si-terminated surface, which is representative for bulk-type Ce, the strong hybridization between Ce 4*f* and Rh 4*d* electrons essentially suppresses the 4*f* ^0^ signal and induces momentum dependent fine structure just below the Fermi level, which reflects the crystal electric field splitting of the magnetic 4*f* ^1^ configuration. These results likely represent the exemplary PES response expected for a Ce-based magnetic Kondo lattice. The fine structure as observed in CeRh_2_Si_2_ indicates the following. First, there is a weakly hybridized excited CEF level at 48 meV. Second, there is a state around 62 meV, but which is spread over a large energy range due to strong hybridization and third, there is a strongly dispersive band stemming from the Γ_7_ ground state.

More importantly, the fact that we observed the CEF satellites in the PES signal taken at *T*∼1 K<*T*_N_ proves that even in the large moment AFM ordered state there is a significant mixing between the *f* ^1^ and the *f* ^0^ state, despite the observation of a small Fermi surface apparently without *f* degree of freedom in dHvA experiments. Thus our ARPES results provide important information on the entanglement between 4*f* and valence states in an AFM-ordered Kondo lattice systems, which is likely difficult to get by other kinds of measurements.

## Methods

### ARPES measurements

ARPES studies were performed at the 1^3^ ARPES instrument of the BESSY-II synchrotron facility. The spectra were acquired using a Scienta R4000 electron energy analyzer. The overall energy and angular resolutions were set to 6 meV and 0.1, respectively. High-quality single-crystalline samples of CeRh_2_Si_2_ were cleaved *in situ* in ultrahigh vacuum at a base pressure better than 8 × 10^−11^ mbar. The sample was kept at a temperature of ∼1 K during the measurements. The photon energy was varied to take advantage of a resonant enhancement of the 4*f* emission cross-section at the Ce 4*d*–4*f* excitation threshold at 121 eV.

### Sample preparation and specific heat measurements

Single crystals were grown by the in-flux technique following the procedure described in detail in ref. 39. Polycrystalline CeRh_2_Si_2_ and LaRh_2_Si_2_ samples were prepared by arc melting of the constituent elements under a high-purity Argon atmosphere. As-cast ingots were annealed for 2 days at 1,200 °C under high vacuum. Chemical composition, crystal structure, and physical properties of single crystals and polycrystals were checked by microprobe, X-ray diffraction and resistivity measurements, respectively. Specific-heat measurements were performed on polycrystalline samples of CeRh_2_Si_2_ and LaRh_2_Si_2_ using the standard specific heat option of the physical property measurement system, Quantum Design, USA. LaRh_2_Si_2_ is here taken as reference compound for subtracting the non-*f* part of the specific heat, that is, the phonon and the non-*f* electron contributions. Since at high temperatures the 4*f* contribution is tiny compared to the phonon part, special care was taken to get accurate absolute values. Both the CeRh_2_Si_2_ and the LaRh_2_Si_2_ samples were measured under identical conditions. For each sample, we determined in a first run the addenda (platform and grease) and then in a second run the total specific heat (addenda+sample). The whole procedure was repeated with a different part of the polycrystalline batches in order to get two independent sets of data. Both sets agreed very nicely, and the 4*f* specific-heat deduced from the these two data sets differ by <0.4 J mol^–1^ K^–1^ in the whole *T* range up to 300 K. The magnetic entropy S_4*f*_ was determined by numerically integrating *C*_4f_/*T* and using a liner extrapolation for the *T* range below 2 K.

We used the analytical expression of Romero *et al*.^37^ to generate *C*_4*f*_ and *S*_4*f*_ for different CEF schemes (Δ_1_ and Δ_2_ energy of excited levels) and different values of the Kondo temperature (*T*_K_) and width of the first excited CEF level (Γ_1_). The values for the different parameters were chosen on the basis of previous and present experimental results (see main text). Desgranges provided us with his numerical results for the Kondo+CEF model for the cases *A*2=*A*4=0.7 *T*_KN6_ and *A*4=0, *A*2=*T*_KN6_ (nomenclature as in ref. 38), which correspond to the cases Δ_2_=2·Δ_1_ and Δ_2_=Δ_1_, respectively. *T*_KN6_ was scaled in order to get the best match for the specific heat data. *T*_K_ (for the ground state doublet) was calculated from *T*_KN6_ by combining the numerical result lim(*C/R*·*T*_KN6_/*T*)_*T*→0_≅20.0 and the definition lim(*C/T*)_*T*→0_=*R*·*π*/(3*T*_K_)^38^.

## Additional information

**How to cite this article:** Patil, S. *et al*. ARPES view on surface and bulk hybridization phenomena in the antiferromagnetic Kondo lattice CeRh_2_Si_2_. *Nat. Commun.* 7:11029 doi: 10.1038/ncomms11029 (2016).
